# Pregnancy-associated breast cancer and increased risk of pregnancy-associated recurrence: a case report

**DOI:** 10.1186/1752-1947-6-144

**Published:** 2012-06-07

**Authors:** Freya Schnabel, Jessica Billig, Arielle Cimeno, Jennifer Chun

**Affiliations:** 1 Department of Surgery, New York University Langone Medical Center, NYU Cancer Institute, New York, NY, USA; 2 New York University Langone Medical Center, School of Medicine, New York, NY, USA

## Abstract

**Introduction:**

Pregnancy-associated breast cancer refers to breast cancer diagnosed during pregnancy, lactation, or within twelve months postpartum. Recent studies suggest that, when matched for age and stage, the prognosis of pregnancy-associated breast cancer is comparable to non-pregnancy-associated breast cancer. However, the risk for breast cancer recurrence associated with subsequent pregnancies in this population is not clear.

**Case presentation:**

We describe the case of a Caucasian woman who was initially treated for pregnancy-associated breast cancer at age 23, three months after the birth of her third child. She underwent a total mastectomy with axillary node dissection, followed by chemotherapy and hormonal therapy. Ten years later, when the patient was 24 weeks pregnant with her fourth child, she presented with an ipsilateral chest wall recurrence of breast cancer. To the best of our knowledge, this represents the first reported case of a pregnancy-associated recurrence in a patient previously treated for pregnancy-associated breast cancer.

**Conclusion:**

The case described here is the first report of a second occurrence of pregnancy-associated breast cancer. This case raises the possibility that pregnancy may represent a unique trigger for breast malignancy in a specific cohort of women. Although there is data showing no increase in the risk of recurrence for women who become pregnant after breast cancer treatment, pregnancy-associated breast cancer may be a distinct clinical category where subsequent pregnancies after treatment may confer an increased risk of recurrent disease.

## Introduction

In 10 % of women diagnosed with breast cancer (BC) under the age of 40 years, the disease is associated with pregnancy [1]. BC is diagnosed in approximately one in 3,000 pregnancies, making it the second most common pregnancy-associated malignancy (after cervical cancer) [2]. With more women delaying childbearing, researchers predict that the prevalence of pregnancy-associated breast cancer (PABC) will continue to rise [2]. Even though increasing parity is protective against BC, women at any age are at a greater risk of developing the disease within two years postpartum [3]. The increased incidence of BC following childbirth suggests that pregnancy may stimulate the growth of cells that have already undergone malignant transformation [3-5]. While the exact mechanisms for this phenomenon are unknown, estrogen and progesterone are established mitogens for breast tissue [5,6]. Several theories have been postulated about the effect of the unique hormonal milieu of pregnancy in promoting the expansion of malignant cells [5,6].

Historically, it was assumed that PABC carried a worse prognosis than BC [7]. PABC usually presents at a more advanced stage, with larger primaries and more lymph node involvement [2,8]. This may be due to the difficulty of detecting BC via physical examination, mammography and ultrasound in pregnant and lactating women [1]. In general, mammography in women of reproductive age usually reveals dense parenchyma, decreasing the sensitivity of the exam. It has now been shown that, when matched for age and stage, the prognosis for PABC and BC is comparable. Once detected, the histology of PABC is similar to cancer in non-pregnant women [1]. Although there is a higher frequency of lymphovascular invasion and high nuclear grade in PABC, these features are frequently found in patients under 40 years and are thought to be more correlated with age than pregnancy [1]. The current management of PABC closely follows the protocols in place for similarly staged BC, and PABC diagnosed after delivery is treated as typical BC [9].

With women delaying childbirth, there is a larger group of premenopausal survivors of BC interested in subsequent childbearing [10]. In the past, women were discouraged from childbearing after BC diagnosis; yet, contemporary research establishes that pregnancy after breast cancer does not imply a worse prognosis. From retrospective trials, pregnancy after BC does not increase risk of recurrence [10], and there is no significant difference in the survival of women who have children after breast cancer treatment and those who do not [4]. Pregnancy after BC treatment implies no further risk, and therefore more breast cancer survivors are choosing to become pregnant.

In the following case, a patient who had been treated for PABC developed another episode of PABC during a subsequent pregnancy ten years after her initial diagnosis. Our case, coupled with the current theories regarding pregnancy and breast malignancy, strongly suggests that women with PABC may represent a specific subgroup of women for whom a subsequent pregnancy may impart unique risks. This observation supports the need to consider a prior history of PABC when counseling BC survivors concerning the potential risks of future pregnancies.

## Case presentation

A 23-year-old Caucasian woman first presented to our department 15 years ago with a palpable mass in her left breast three months after the delivery of her third child. At the time, she was breast-feeding. She had no family history of breast or ovarian cancer, and no personal history of breast disease. Menarche occurred at age thirteen, and she was nineteen when she delivered her first child. In view of her young age and Ashkenazi Jewish heritage, she subsequently underwent genetic testing, which was negative for *BRCA1* and *BRCA2* mutations. At the time of her initial presentation, an ultrasound of her left breast revealed a 1.7 cm mass in the area of palpable abnormality, and ultrasound guided fine needle aspiration (FNA) was suspicious for carcinoma. Extensive microcalcifications in her left breast were demonstrated on mammography. A left breast lumpectomy showed extensive intraductal and invasive ductal carcinoma with multifocally involved margins. The largest single focus of invasive cancer measured 1.5 cm. The cancer was 38 % estrogen receptor positive and 35 % progesterone receptor positive. Our patient then underwent a left total mastectomy with axillary lymph node dissection and transverse rectus abdominis myocutaneous flap reconstruction. The pathology at the time revealed additional extensive intraductal carcinoma, and axillary nodes showed no evidence of disease. Postoperatively, she received chemotherapy with cyclophosphamide, doxorubicin (Adriamycin) and 5-fluorouracil and was maintained on tamoxifen and leuprolide for three years. She was monitored closely and was without evidence of recurrence for approximately 10 years.

Five years ago, our patient was evaluated with mammography, ultrasound and magnetic resonance imaging, which showed no evidence of malignancy in the contralateral breast. Our patient returned six months later, 20 weeks pregnant, at which time her clinical exam was unremarkable. One month later, our patient presented in the 24th week of pregnancy with a palpable mass in the upper-outer portion of her reconstructed left breast. On examination, a subcutaneous mass was appreciated, associated with the native skin of her reconstructed left breast. FNA of the mass identified malignant cells. Our patient underwent wide local excision of the mass. Histology confirmed poorly differentiated breast carcinoma invading into adipose tissue. The tumor, measuring 0.4 cm in its widest dimension, was noted to be estrogen receptor negative, but highly progesterone receptor positive (95 %) with overexpression of human epidermal growth factor receptor 2/Neu. After delivery of a healthy male child at term, our patient was treated with tamoxifen and trastuzumab and underwent radiation therapy to her left chest wall. At present, she remains on tamoxifen and is doing well without recurrent disease.

## Discussion

Historically, women were counseled against pregnancy after BC treatment owing to concerns regarding increasing risk for recurrence and mortality. Our increased understanding of the hormone responsiveness of BC produced the theory that the elevated levels of estrogen and progesterone in pregnancy could increase the risk of breast cancer recurrence. However, contemporary data shows no increased risk of recurrence and no difference in survival in women who had children after BC therapy when compared with women who chose not to become pregnant [2,10,11]. As a result, BC is no longer considered an absolute contraindication for subsequent pregnancies. Some recent literature recommends counseling patients to wait two years after diagnosis and treatment before attempting to become pregnant, as two years represents the median time to recurrence of disease [2,11]. Additionally, a study by Clark and Reid of 330 patients concluded that women who waited two years after BC treatment to conceive had a significantly increased five-year survival rate compared with those who waited six months to conceive [12]. While pregnancy appears to confer no increased risk for recurrent BC, there is a lack of data regarding outcomes of subsequent pregnancy for women with initial PABC.

At this point, it is possible that there are insufficient numbers of survivors of PABC who have subsequently become pregnant to establish any associated increase in risk for recurrence. This case report illustrates a recurrence of PABC that, to the best of our knowledge, has not previously been described in the medical literature. In this case, the time lag of 10 years indicates that the second episode of PABC is likely to be a second primary. This is supported by the slightly different biomarker profile. In our patient, who lacks an identified genetic predisposition to BC, the unique hormonal milieu of pregnancy may have been a specific trigger for malignancy.

## Conclusion

In the modern era, we have come to recognize that women can bear children after BC treatment without compromising their survival. There is insufficient data to assess the impact of additional pregnancies on the risk of recurrence and survival for women with PABC. The case in this report indicates that PABC survivors, unlike BC survivors, may be at risk for recurrent disease during subsequent pregnancies. They may be uniquely susceptible to the hormonal milieu of pregnancy, producing an increased risk for recurrence with subsequent pregnancies. In the absence of specific information regarding this subgroup of women, caution should be exercised when counseling survivors of PABC regarding subsequent pregnancy. With this understanding, we suggest that women who choose to become pregnant after PABC should be monitored more closely during pregnancy and postpartum. Longitudinal studies will be important to establish the BC-related outcomes of women who become pregnant after treatment of PABC. Further research is needed to elucidate the underlying mechanisms and specific risks, and allow for appropriate evidence-based counseling of survivors of PABC.

## Consent

Written informed consent was obtained from the patient for publication of this case report. A copy of the written consent is available for review by the Editor-in-Chief of this journal.

## Abbreviations

BC, Breast cancer; FNA, Fine needle aspiration; PABC, Pregnancy-associated breast cancer.

## Competing interests

The authors declare that they have no competing interests.

## Authors’ contributions

FS is the patient’s surgeon and was a major contributor in writing and editing the manuscript. JB and AC performed the medical literature review on the topic of PABCs and were also major contributors in writing the manuscript. JC reviewed the statistics and was a major contributor in designing, writing and editing the manuscript. All authors read and approved the final manuscript.

## References

[B1] Barnes DM Newman LA Pregnancy-associated breast cancer: a literature review Surg Clin North Am 2007 87 2 417 430 10.1016/j.suc.2007.01.008 17498535

[B2] Molckovsky A Madarnas Y Breast cancer in pregnancy: a literature review Breast Cancer Res Treat 2008 108 333 338 10.1007/s10549-007-9616-6 17530426

[B3] Lambe M Hsieh CC Trichopoulos D Ekbom A Pavia M Adami HO Transient increase in the risk of breast cancer after giving birth N Engl J Med 1994 331 1 5 9 10.1056/NEJM199407073310102 8202106

[B4] Kroman N Jensen MB Wohlfahrt J Ejlertsen B Danish Breast Cancer Cooperative Group Pregnancy after treatment of breast cancer – a population based study on behalf of Danish Breast Cancer Cooperative Group Acta Oncol 2008 47 4 545 549 10.1080/02841860801935491 18465320

[B5] Fornetti J Martinson H Borges V Schedin P Emerging targets for the prevention of pregnancy-associated breast cancer Cell Cycle 2012 11 4 639 640 10.4161/cc.11.4.19358 22374663 PMC3318100

[B6] Schedin P Pregnancy-associated breast cancer and metastasis Nat Rev Cancer 2006 6 4 281 291 10.1038/nrc1839 16557280

[B7] Ali SA Gupta S Sehgal R Vogel V Survival outcomes in pregnancy associated breast cancer: a retrospective case control study Breast J 2012 18 2 139 144 10.1111/j.1524-4741.2011.01201.x 22356297

[B8] Johansson AL Andersson TM Hsieh CC Cnattingius S Lambe M Increased mortality in women with breast cancer detected during pregnancy and different periods postpartum Cancer Epidemiol Biomarkers Prev 2011 20 9 1865 1872 10.1158/1055-9965.EPI-11-0515 21750168

[B9] Sukumvanich P Review of current treatment options for pregnancy-associated breast cancer Clin Obstet Gynecol 2011 54 1 164 172 10.1097/GRF.0b013e3182083a44 21278516

[B10] Upponi SS Ahmad F Whitaker IS Purushotham AD Pregnancy after breast cancer Eur J Cancer 2003 39 6 736 741 10.1016/S0959-8049(02)00870-5 12651197

[B11] Azim HA Santoro L Pavlidis N Gelber S Kroman N Azim H Peccatori FA Safety of pregnancy following breast cancer diagnosis: a meta-analysis of 14 studies Eur J Cancer 2011 47 1 74 83 10.1016/j.ejca.2010.09.007 20943370

[B12] Clark R Reid J Carcinoma of the breast in pregnancy and lactation Int J Radiat Oncol Biol Phys 1978 4 7–8 693 698 711539 10.1016/0360-3016(78)90196-7

